# Microwave induced synthesis of graft copolymer of binary vinyl monomer mixtures onto delignified *Grewia optiva* fiber: application in dye removal

**DOI:** 10.3389/fchem.2014.00059

**Published:** 2014-08-11

**Authors:** Vinod Kumar Gupta, Deepak Pathania, Bhanu Priya, Amar Singh Singha, Gaurav Sharma

**Affiliations:** ^1^Department of Chemistry, Indian Institute of Technology RoorkeeRoorkee, India; ^2^Department of Chemistry, Shoolini University of Biotechnology and Management SciencesSolan, India; ^3^Department of Applied Chemistry, National Institute of Technology HamirpurHamirpur, India

**Keywords:** comonomer, microwave, delignified *Grewia optiva* fiber, physicochemical properties, methylene blue

## Abstract

Grafting method, through microwave radiation technique is very effective in terms of time consumption, cost effectiveness and environmental friendliness. Via this method, delignified *Grewia optiva* identified as a waste biomass, was graft copolymerized with methylmethacrylate (MMA) as an principal monomer in a binary mixture of ethyl methacrylate (EMA) and ethyl acrylate (EA) under microwave irradiation (MWR) using ascorbic acid/H_2_O_2_ as an initiator system. The concentration of the comonomer was optimized to maximize the graft yield with respect to the primary monomer. Maximum graft yield (86.32%) was found for dGo-poly(MMA-co-EA) binary mixture as compared to other synthesized copolymer. The experimental results inferred that the optimal concentrations for the comonomers to the optimized primary monomer was observed to be 3.19 mol/L × 10^−1^ for EMA and 2.76 mol/L × 10^−1^ for EA. Delignified and graft copolymerized fiber were subjected to evaluation of physicochemical properties such as swelling behavior and chemical resistance. The synthesized graft copolymers were characterized with Fourier transform infrared spectroscopy (FTIR), scanning electron microscopy (SEM), thermogravimetric analysis (TGA) and X-ray diffraction techniques. Thermal stability of dGo-poly(MMA-co-EA) was found to be more as compared to the delignified *Grewia optiva* fiber and other graft copolymers. Although the grafting technique was found to decrease percentage crystallinity and crystallinity index among the graft copolymers but there was significant increase in their acid/base and thermal resistance properties. The grafted samples have been explored for the adsorption of hazardous methylene dye from aqueous system.

## Introduction

Natural biomasses such as natural fibers are utilized by humans for household or other conventional applications (Necula et al., 2010; Ramanaiah et al., 2011a; Sharma et al., 2013). However, during the past few decades, natural polymers have found various applications in different fields such as building materials, sports equipment, automobiles, electrolytes, energy storage, aerospace, and as adsorbent for toxic metal ion from different resources (Kiani et al., 2011; Ramanaiah et al., 2011b; Sis et al., 2013).

The wider applicability of natural fibers has been due to the exhibition of diverse properties like low density, low health hazards, biodegradability, better wear resistance, and a high degree of flexibility, low cost, renewability, and high specific strength. These fibers have been found to be sensitive to moisture, chemicals, water, and their properties are consequently degraded when they come in contact with harsh environmental conditions. A variety of chemical treatments and modifications have been employed onto natural fibers to enhance their application in different areas including composite materials; thereby necessitating the improvement of their existing properties (Singha and Rana, 2010a).

Graft copolymerization of vinyl monomers onto natural and synthetic polymers has the advantage of incorporation of additional properties of the monomer. A considerable number of studies on graft copolymerization of single monomers onto cellulose using different methods of initiation have been reported. But graft copolymerization of binary mixtures of vinyl monomers has special importance in comparison to simple grafting of individual monomers. This technique of grafting of monomer mixtures has the advantage of creating grafted chains with tailor made properties for specific applications. The synergistic effect of the comonomers in grafting mixtures plays an important role in controlling the composition and graft yield onto cellulose (Singha et al., 2013; Thakur et al., 2014).

Graft copolymerization of methyl methacrylate (MMA) onto cellulose by chemical and radiation methods is well investigated (Singha et al., 2014). Microwave irradiation (MWR) gives rapid energy transfer and high-energy efficiency. Microwave radiation (MWR) assists in direct heating of solvents and reactants. Owing to this interesting heating mechanism, which is clearly different from other conventional heating, selective heating can be accomplished and many reactions can be accelerated (Mansour and Nagaty, 1975). MWR technique reduces the extent of physicochemical stresses to which the fibers are exposed during the conventional techniques. Microwave technology uses electromagnetic waves, which passes through material and causes its molecules to oscillate. Microwave energy is not observed by non-polar materials to any degree while polar water molecules held within a polymer matrix do absorb energy very proficiently, thus becoming heated (Kaith and Kalia, 2008a). A few workers have studied the grafting of vinyl monomers onto various natural polymers under MWRs inorder to improve the properties of the backbone polymer (Kaith and Kalia, 2007, 2008b).

The grafting with binary mixture of monomers provides an opportunity to prepare the material for specific applications (Singha and Rana, 2010b, 2012a). However, in these investigations no systematic analysis of grafting parameters has been reported, as has been carried out using binary mixture of monomers. In these investigations, the concentration dependant monomer-monomer interactions in the reaction mixture have been found responsible for controlling the graft yield and the composition of the grafted chains. Because of synergistic effect of the added comonomer, the graft yield and other grafting parameters have shown improvement, in comparison with graft copolymerization carried out with individual monomers. Investigations have revealed that grafted chains of desired properties can be obtained by using suitable combination of monomers and their compositions in the feed (Kitagawa and Tokiwa, 2006; Singha and Rana, 2012b,c).

Lignocellulosic fibers have drawn considerable attention for environment protection as they are abundant in nature, inexhaustible, inexpensive, renewable, stable, hydrophilic, biodegradable and modifiable biopolymers. The fibers are generally composed of cellulose embedded in a matrix of other structural biopolymers like hemicelluloses, lignin, pectin, waxy substances, nitrogen-containing substances, minerals, organic acids etc. Cellulose is the highly functionalized, linear stiff chain homopolymer. It is known to be hydophillic, biodegradable and has broad chemical modifying capacity. These materials have been used in many industrial applications and as an adsorbent due to their heterogeneous nature (Jonathan and Chen, 2010; Kalia and Averous, 2011). Therefore, efforts have been made to convert this biomass into inexpensive and effective adsorbent.

Methylene blue is known to cause problems in humans ranging from nausea, vomiting, profuse sweating, mental disorders, hemoglobinemia or blue baby syndrome etc. (Cowling, 1975; Lynd et al., 1999). Hence, wastewater containing dyes need at source remediation before being discharged into natural water bodies. Many physiochemical methods like coagulation, flocculation, ion exchange, membrane separation, photodegradation, electrochemical oxidation etc. have been used for the treatment of contaminated water (Bhattacharyya and Sharma, 2005; Wang and Zhu, 2007; Gupta et al., 2013b,c, 2014; Pathania and Rathore, 2014). Among these methods, adsorption is known to be efficient, and is widely used in wastewater treatment because of its simplicity, economic viability, technical feasibility, and social acceptability. This has led to the development of cheaper, effective, easily available and biodegradable materials for the adsorption of pollutants from water system. Several researches have made significant contributions in this area, utilizing a number of natural materials like chitosan, pectin, rice-husk, mango seed kernel powder, peanut hull, cross-linked chitosan beads, natural fibers etc. for the removal of the dyes from water (Pathania and Sharma, 2012; Gupta et al., 2013a).

In the present study, efforts have been made to modify the surface of deilgnified *Grewia optiva* fiber through graft copolymerization method of binary monomer mixture of MMA-co-EMA and MMA-co-EA using ascorbic acid (ASC)/H_2_O_2_ redox initiator system with MMA as the principal monomer. Fiber was extracted from the *Grewia optiva* tree and delignified by chemical method. *Grewia optiva* fiber had a composition of approximately 58–62% cellulose, 22–24% hemicelluloses, and 14–16% lignin. Its composition showed a dependence on the source, age and separating techniques. The extracted fibers due to their excellent mechanical properties are utilized by villagers for domestic purposes in the making of ropes, bags, mats etc. These fibers have excellent potential as textile fiber and are a promising candidate as fiber reinforcement for polymer matrix based composites. The potential of the grafted fiber for the removal of methylene blue dye from water system was also explored.

## Experimental

### Materials and methods

The monomers, MMA, ethyl acrylate (EA) and ethyl methacrylate (EMA) were received from S.D. Fine, India and used as received. Acetone of 99% purity supplied by Rankem, India was used for removal of homopolymer. ASC and hydrogen peroxide (H_2_O_2_) were supplied by E. Merck Pvt. Ltd., India, used as initiator, Sodium chlorite (NaClO_2_) (Himedia Pvt. Ltd., India) was used as received. Weighing of the samples was done on Libror AEG-220 (Shimadzu, Japan) electronic balance. Microwave equipment (Grill Microwave Oven 20PGI) was used for graft copolymerization.

#### Delignification of Grewia optiva fiber

*Grewia optiva* tree branches were collected from Shivalik region of Himachal Pradesh, India. After collection, these branches were immersed in continuously flowing fresh water for 30 days at temperature between 25 and 30°C. The branches were then taken out of water and fibers were gently separated from sticks by dissolving the cementing and gummy material through beating. The obtained fibers were washed with detergent to remove the impurities. The fibers were then dried in a hot air oven maintained at 80°C for 12 h. Fibers were treated with 0.7% NaClO_2_ at pH 4 and maintained to liquor ratio of 1:50. This mixture was boiled for 2 h with continuous stirring. After treatment, the fibers were washed and dried at 80°C for 72 h. The treated fibers were designated as delignified *Grewia optiva* fiber. The delignified *Grewia optiva* fibers were then cut into pieces of length 85–100 mm and 500 mg of the fibers was used for grafting reaction.

#### Microwave radiations induced grafting onto delignified Grewia optiva fiber

Initially optimization of different reaction parameters such as reaction time, microwave power, initiator concentration and monomer concentration was carried out for graft copolymerization of the principal monomer MMA onto delignified *Grewia optiva* fiber backbone prior to carry out grafting with a binary mixture under MWR radiation. Delignified *Grewia optiva* fiber (500 mg) was immersed in 100 ml of distilled water for 24 h prior to its grafting under the influence of MWRs. A definite amount of ASC (3.74 mol/L × 10^−2^), H_2_O_2_ (0.97 mol/L × 10^−1^), and monomer (1.87 mol/L × 10^−1^) was added to the reaction flask. The co-monomers were then added to the reaction mixture with concentration ranging from 2.39 to 3.99 × 10^−1^ mol/L for EMA and 1.84 to 3.68 × 10^−1^ mol/L for EA under continuous stirring. The mixture was then subjected to MWR for 10 min at MWR power of 110 W to get maximum percent graft yield. The reaction was stopped after 10 min and the graft copolymer obtained was taken out and subjected to the removal of homopolymer formed during the grafting reaction.

Percent graft yield (% *P_g_*), percent graft efficiency (% *P_e_*), percent monomer conversion (%C), and percent homocopolymers (% *H_cp_*) were calculated by following methods (Kaith and Kalia, 2007).

(1) $$\%\;Grafting\;\left(p_{g}\right)\;=\;\frac{W_{g}\;-W}{W}\;\times \;100$$

(2) $$\%\;Efficiency\;\left(p_{e}\right)\;=\frac{W_{g}\;-W}{W_{m}}\;\times \;100$$

(3) $$\%\;Homocopolymer\;\left(\%\;H_{cp}\right)\;=\;\frac{W_{Hcp}}{W_{m}}\;\times \;100$$

where *W*, *W_g_*, *W_m_*, and *W_Hcp_* are the weights of delignified fiber, grafted fiber, monomer and homocopolymers.

#### Swelling behavior

The swelling behavior of the grafted and ungrafted fibers was studied in polar and non-polar solvents such as water, n-butanol, DMSO and carbon tetrachloride. Dry samples of grafted and ungrafted fibers were subjected to the evaluation of swelling behavior by immersing the known weights of the fibers in certain amounts of different solvents for 24 h. The samples were then taken out and excess solvent was removed by pressing between the folds of the filter paper. The samples were weighed again to obtain the final weight. The degree of swelling was calculated by using the following formula (Singha and Rana, 2012c):

(4) $$\%\;Swelling\;=\;\frac{W_{f}\;-\;W_{i}}{W_{i}}\;\times \;100$$

where, *W_i_* is the initial weight of the dried fiber and *W_f_* is the final weight after the swelling.

#### Acid and base resistance

The acid and base resistance of the fibers was studied as a function of percentage weight loss when treated with different chemicals. A known amount (*W_i_*) of the ungrafted and grafted fibers was separately treated with a fixed volume of hydrochloric acid and sodium hydroxide of different strengths for 24 h. The fibers were then washed 2–3 times with distilled water and finally dried in an oven at 70°C to a constant weight. The samples were weighed again to obtain the final weight (*W_f_*). The percentage of weight loss was determined by using the following formula (Kitagawa and Tokiwa, 2006).

(5) $$\%\;Weight\;loss\;=\;\frac{W_{i}\;-W_{f}}{W_{i}}\;\times \;100.$$

### Characterization

#### Scanning electron microscopy (SEM)

Scanning electron microscopic studies of raw, delignified and grafted *Grewia optiva* fibers were carried out on Quanta FEG 450 electron microscope. All the samples were gold plated to make them conducting. The images were taken at a resolution of 1000×.

#### X-ray diffraction studies (XRD)

X-ray diffraction studies were performed on a Philip 1710 X-ray diffractometer using CuKα (1.5418 A) radiation, a Ni-Filter and a scintillation counter as a detector at 40 kV and 40 mA on rotation from 2 to 60° at 2θ scale. The counter reading of peak intensity close to 22 and 15° represents the crystalline and amorphous materials in cellulose, respectively. The percentage crystallinity (%Cr) and crystallinity index (C.I.) were calculated according to Equations (6, 7) as follow (Singha and Rana, 2012c):

(6) $$\%\;Cr\;=\;\frac{I_{22}}{I_{22}\;+\;I_{15}}\;\times \;100$$

(7) $$C.I.\;=\;\frac{I_{22}\;-\;I_{15}}{I_{22}}$$

#### Fourier transforms infrared studies (FTIR)

Fourier transform infrared spectra of raw *Grewia optiva* fiber, delignified *Grewia optiva* fiber, dGo-poly(MMA), dGo-poly(MMA-co-EA), and dGo-poly(MMA-co-EMA) were recorded on Perkin Elmer FT-IR spectrophotometer using KBr pellets. The samples were exposed to IR radiations in the range of 4000 to 400 cm^−1^ with a resolution of 2 cm^−1^.

#### Thermogravimetric analysis

Thermogravimetric analysis of the raw *Grewia optiva* fiber, delignified *Grewia optiva* fiber, dGo-poly(MMA), dGo-poly(MMA-co-EA), and dGo-poly(MMA-co-EMA) were performed using EXSTAR TG/DTA 6300 at a heating rate of 10°C/min under nitrogen atmosphere. The temperature ranged from 25 to 800°C.

### Dye adsorption study

The adsorption experiments were carried out using simple batch process. In these experiments, 0.1 g of raw, dGo-poly(MMA), dGo-poly(MMA-co-EA), and dGo-poly(MMA-co-EMA) fibers were used as adsorbent and kept in 50 mL of methylene dye solution for adsorption at 35°C. The initial methylene blue dye concentrations were varied between 5 and 30 mgL^−1^. The resultant reaction mixture was stirred for 60 min and amount of dye adsorbed was determined. The amount of dye adsorbed q_e_ (mg g^−1^) was calculated by using following formula:

$${\text{q}}_{\text{e}}\;=\;\frac{\left({\text{C}}_{0}\;-{\text{ C}}_{\text{e}}\right)\text{V}}{\text{M}}$$

where C_0_ is the initial concentration of dye, C_e_ is the concentration time at equilibrium in solution, V is the volume and M is the adsorbed mass.

## Results and discussions

### Mechanism

**Scheme 1:**
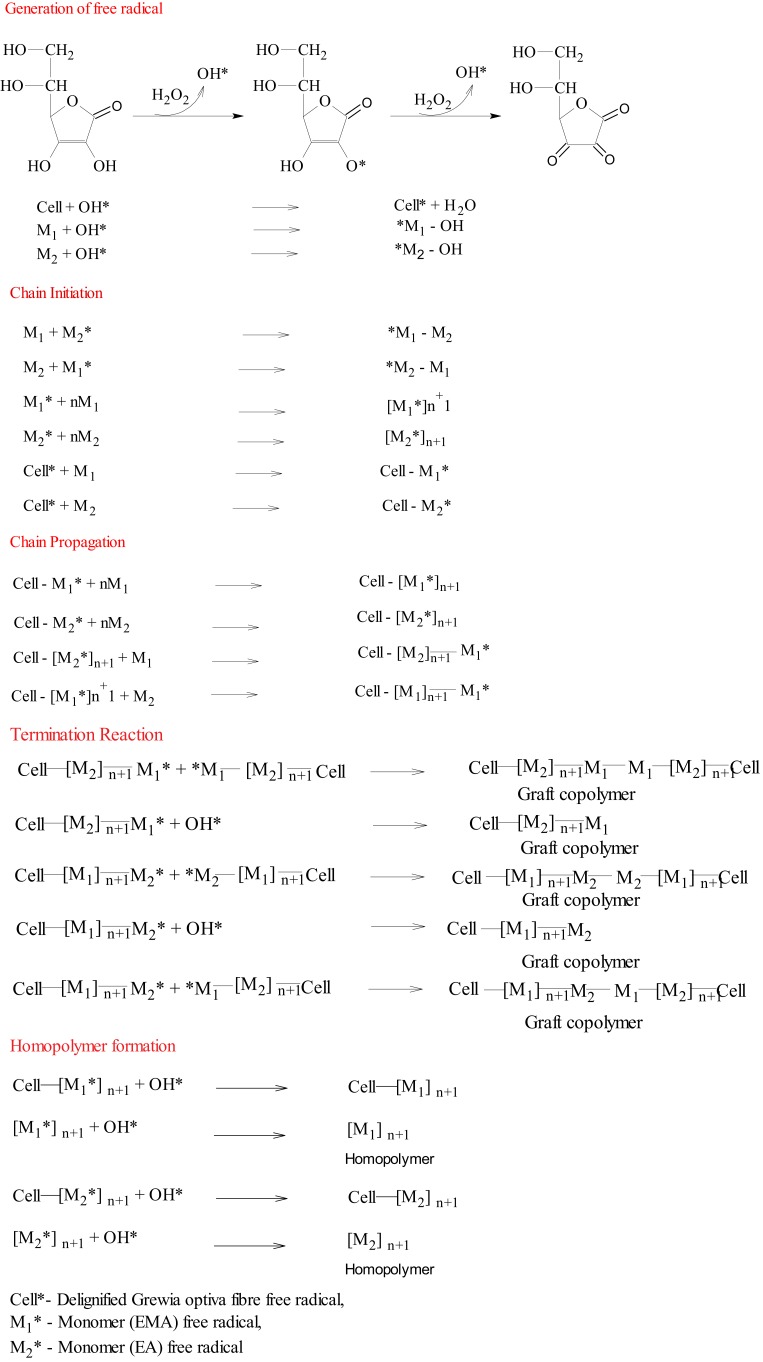
**Probable mechanism of graft copolymerization of binary monomer mixture**.

The interaction of ASC with H_2_O_2_ generates OH^*^ (Scheme 1) (Kitagawa and Tokiwa, 2006), and these free radicals are known to be responsible for free radical generation on polymer backbone and monomer as well as for further chain propagation, thereby resulting in the formation of a graft copolymer along with a homopolymer. On the other hand, MWR also produces free radicals on polymeric backbone and monomer, which can be explained through the following mechanism:

### Effect of the concentration of binary vinyl monomer mixtures on percentage grafting

Initially optimizations of different reaction parameters were carried out under MWR irradiation for graft copolymerization of MMA onto backbone. The experimental results showed that the optimal conditions for grafting were: exposure time—10 min, microwave power—110 W, ASC conc.—3.74 mol/L × 10^−2^, H_2_O_2_ conc.—0.97 mol/L × 10^−1^, monomer conc.—1.87 mol/L × 10^−1^. The maximum *P_g_* of dGo-poly(MMA) was found to be (26.54%). Graft copolymerization of MMA-co-EMA (ethyl methacrylate) and MMA-co-EA (ethyl acrylate) binary mixtures onto delignified *Grewia optiva* fiber under optimized reaction conditions using MMA—1.87 mol/L × 10^−1^ the principal monomer and EMA—3.19 mol/L × 10^−1^ showed 51.56%; and MMA—3.19 mol/L × 10^−1^ and EA—2.76 mol/L × 10^−1^ showed 86.32% (Table 1). Higher percentage graft yield observed in the case of binary vinyl mixtures could be explained on the basis of the monomer reactivity ratio. The reactivity ratios in the case of different binary vinyl monomer mixtures have been found to be MMA-co-EMA: *r*_1_ = 2.15, *r*_2_ = 0.42; MMA-co-EA: *r*_1_ = 1.322, *r*_2_ = 0.138 (Brandrup, 1975). The formation of copolymer between the two different monomeric moieties takes place, which suppresses the comonomer homopolymerization due to small *r*_2_ values in all the binary mixtures. On the other hand *r*_1_ values clearly indicate the formation of a copolymer between the different monomers in the binary mixtures, which suppresses the formation of principal monomer homopolymerization, which resulting in higher graft yields. In the case of principal monomer, lower graft yield could be due to the higher reactivity ratio of MMA with MMA which result in more homopolymerization. Higher percentage graft yield obtained in case of MMA-co-EA (86.32%) than in the case of MMA-co-EMA (51.56%) binary mixtures which is higher than grafting of MMA (26.54%). It could be due to the more reactivity of EA that MMA and EA radical is less hysterical hindered. Then it can form more graft sites. The low graft yield with MMA-co-EMA binary mixture was due to the less reactivity of EMA then EA.

**Table 1:** **Evaluation of optimum reaction parameter for grafting of binary vinyl monomer mixture onto delignified *Grewia optiva* fiber**.

**Sr. No**	**× 10^−1^ mol/L**	**P_g_(%)**	**P_e_(%)**	**%H_cp_**
**MMA + EMA**				
1	1.87 + 2.39	33.62	6.14	19.63
2	1.87 + 2.79	44.68	6.99	20.43
3	**1.87** + **3.19**	**51.56**	**7.06**	**22.84**
4	1.87 + 3.59	44.16	5.38	20.13
5	1.87 + 3.99	29.74	3.26	17.38
**MMA + EA**				
6	1.87 + 1.84	40.38	10.93	17.26
7	1.87 + 2.31	55.28	11.98	18.34
8	**1.87** + **2.76**	**86.32**	**15.58**	**20.73**
9	1.87 + 3.22	64.18	9.93	19.35
10	1.87 + 3.68	36.72	4.97	16.67

Optimal conditions for grafting: exposure time = 10 min, microwave power = 110 W, ASC conc. = 3.74 mol/L × 10^−2^, H_2_O_2_ conc. = 0.97 mol/L × 10^−1^ (Singha et al., 2014).

### Chemical resistance

**Figure 1:**
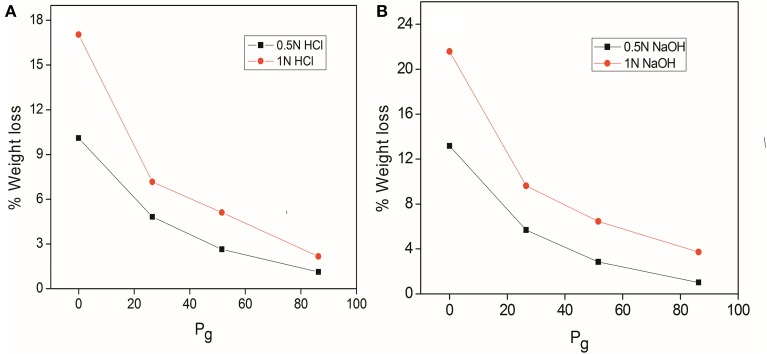
**(A) Acid resistance (B) base resistance of delignified *Grewia optiva* fibre, dGo-poly(MMA), dGo-poly(MMA-co-EA), and dGo-poly(MMA-co-EMA) at maximum *P*_g_**.

Chemical resistance of delignified, MMA grafted fibers, dGo-g-poly(MMA-co-EMA) and dGo-g-poly(MMA-co-EA) samples was studied in HCl and NaOH of different normalities at 0.5 and 1.0 N for 24 h as shown in Figures 1A,B. It has been evident that grafted fibers were more resistant to the attack of acids/base and hence lesser weight loss occurred as compared to ungrafted fibers.

### Swelling studies

**Figure 2:**
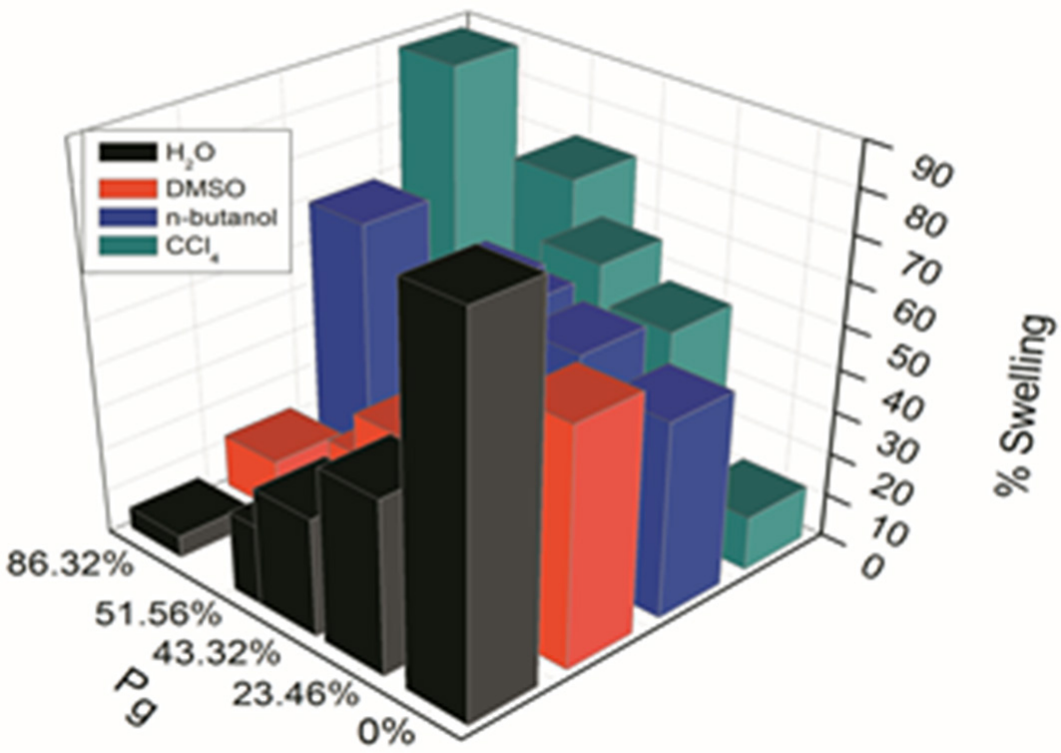
**Swelling behavior of delignified *Grewia optiva* fiber, dGo-poly(MMA), dGo-poly(MMA-co-EA), and dGo-poly(MMA-co-EMA) at maximum *P_g_* in different solvents**.

Figure 2 Shows the swelling behavior of delignified *Grewia optiva* fiber, dGo-poly(MMA), dGo-poly(MMA-co-EA), and dGo-poly(MMA-co-EMA) at *P_g_* 0, 26.54, 86.32, 51.56%, respectively were performed in water, n-butanol, DMSO and CCl_4._ The swelling behavior of ungrafted fiber in different solvents followed the trend as H_2_O > DMSO > n-butanol > CCl_4_. Where as in grafted fiber it varied with *P_g_* and followed the trend as CCl_4_ > n-butanol > DMSO > H_2_O. Delignified *Grewia optiva* fiber possesses hydrophilic -OH groups at C2, C3, and C6 of the glucose unit, and hence has strong affinity with water as compared to other solvents. In case of grafted fibers containing poly(MMA), poly(MMA-co-EMA), and poly(MMA-co-EA) monomer chains, the extent of interaction with water and alcohols is different as compared with ungrafted fibers. This may be due to the blockage of active sites on the main polymeric backbone by poly(MMA), poly(MMA-co-EMA), and poly(MMA-co-EA) chains which resulted in the sorption behavior with different solvents. Further poly(MMA), poly(MMA-co-EMA), and poly(MMA-co-EA) chains on grafted fiber is more solvolysed by non-polar solvent (CCl_4_) than by polar aprotic solvent (DMSO) as compared to that with water or alcohol. Hence more swelling is observed in CCl_4_ as compared to that with other solvents.

### SEM analysis

**Figure 3:**
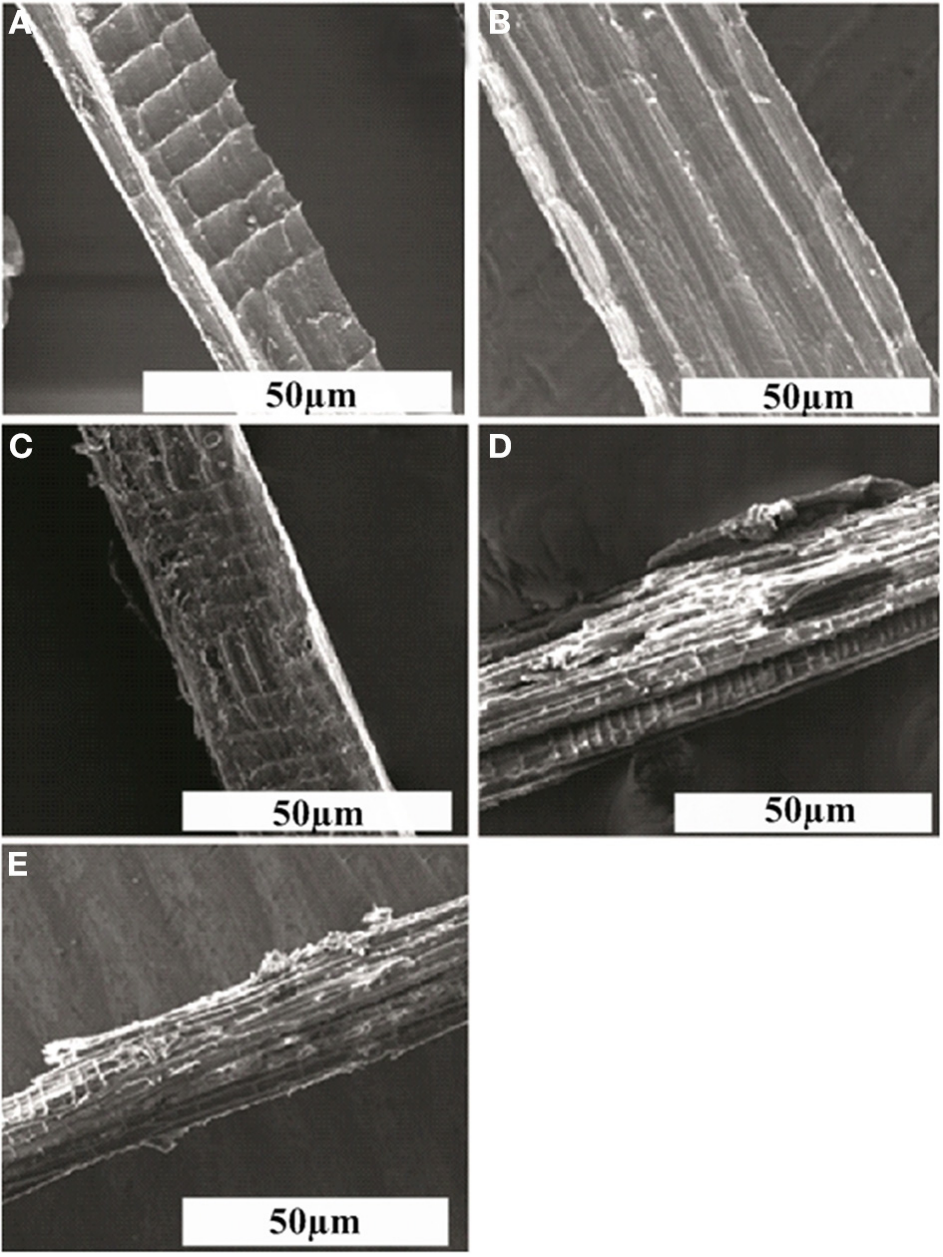
**Scanning electron micrographs of **(A)** raw *Grewia optiva* fibre, **(B)** delignified *Grewia optiva* fibre, **(C)** dGo-g-poly(MMA), **(D)** d*Go*-g-poly(MMA-co-EMA), and **(E)** d*Go*-g-poly(MMA-co-EA)**.

The scanning electron micrographs of raw, delignified and poly (MMA) grafted delignified *Grewia optiva* fibers show a clear cut distinction in, Figures 3A–E. This provides a strong evidence for the change in the surface morphology of the fibers as a result of delignification and grafting of the monomer onto the cellulosic backbone. It is quite evident from the micrographs that delignified fibers (Figure 3B) are smoother than raw fiber (Figure 3A) and surface of graft copolymerized fibers (Figures 3C–E) is visibly rough due to the incorporation of polymeric chains onto the fiber backbone (Singha et al., 2014). This change in morphology ultimately causes changes in the properties of the raw *Grewia optiva* fibers onto graft copolymerization.

### FTIR analysis

**Figure 4:**
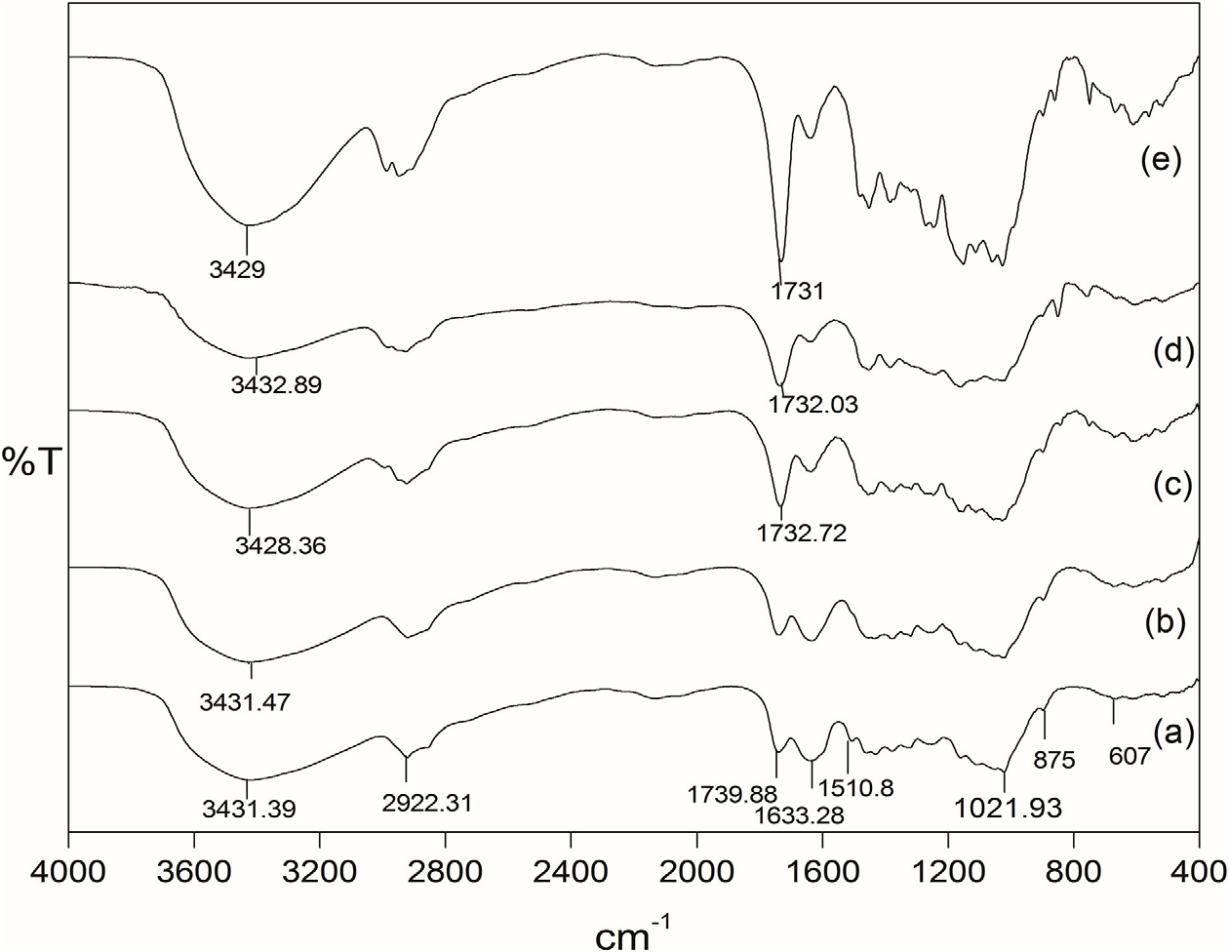
**FTIR spectra of (A) raw *Grewia optiva*, (B) delignified *Grewia optiva* fibre, (C) dGo-poly(MMA), (D) dGo-poly(MMA-co-EA), and (E) dGo-poly(MMA-co-EMA)**.

The comparison of the FTIR spectra of raw, delignified, and MMA grafted *Grewia optiva* fibers has been reported earlier (Singha et al., 2014). Figures 4A–E shows the FT-IR spectrum of raw, delignified, MMA grafted fibers, dGo-g-poly(MMA-co-EMA), and dGo-g-poly(MMA-co-EA). FT-IR spectrum of raw *Grewia optiva* fiber (Figure 4A) shows a broad peak at 607.25 cm^−1^ (due to out of plane –OH bending), 875 cm^−1^ (due to β-glycosidic linkage), 1021.93 cm^−1^ (due to stretching of C-O and -OH), peaks at 1431.01–1451.99 cm^−1^ (due to -CH, -CH_2_, and –CH_3_ bending), 1510.8 cm^−1^ (lignin aromatic ring vibration and stretching). The peak due to lignin at 1510.8 cm^−1^ is significantly disappeared in delignified *Grewia optiva* fiber (Figure 4B). The peaks are observed at 1633.27 cm^−1^ (due to H-O-H bending of absorbed water and for lignin C-H deformation), 2922.31 cm^−1^ (for C-H stretching vibration of aliphatic methylene groups), and 3431.39 cm^−1^ (due to bonded -OH group) in all spectra. An additional peak at 1734.94 cm^−1^ (Figure 4C) is observed due to the carbonyl group (>C=O) of MMA. This confirms the grafting of MMA onto delignified *Grewia optiva* fiber. poly(MMA-co-EMA) and poly(MMA-co-EA) copolymers grafted onto fiber has shown absorption bands at 1732.03 cm^−1^ and 1731 cm^−1^ which correspond to ester carbonyl group of EMA and EA(Figures 4D,E) (Gupta et al., 2002; Gupta and Khandekar, 2006).

### Crystallinity of copolymers

It is evident from Table 2 that raw *Grewia optiva* fiber and delignified *Grewia optiva* fiber showed 66.15 and 71.48% crystallinity. In case of delignified *Grewia optiva*, the incorporation of monomers chains to fiber backbone had impaired the crystallinity of fiber. %Cr decreased rapidly with reduction in its stiffness and hardness (Singha and Rana, 2012c). Whereas, graft copolymers prepared under the influence of MWRs showed fewer disturbances in the crystalline lattice. This is due to the reason that optimum reaction time for grafting under MWR was very low and thus the fiber underwent a fewer disturbances in its crystalline structure. Moreover, fiber faces less surface deformations during grafting process under the influence of MWRs, thereby retaining better crystalline structure. dGo-g-poly(MMA-co-EMA) and dGo-g-poly(MMA-co-EA) showed the %Cr values 54.41 and 51.38, respectively. The grafted fibers show lower percent crystallinity (%Cr) as well as C.I. Lower crystallinity index of the grafted fiber indicates that there may be the disorientation of the cellulose crystals when poly(MMA-co-EA) and poly(MMA-co-EMA) chains are incorporated in the fiber.

**Table 2:** **Percentage crystallinity (%Cr) and crystalline index (C.I.) of raw, delignified and graft copolymers prepared under the influence of MWR**.

**Sample name**	**P_g_**	**At 2θ scale**		**% Cr**	**C.I.**
		**I_22_**	**I_15_**		
Raw *Grewia optiva* fiber	–	903	432	67.64	0.52
Delignified *Grewia optiva* fiber	–	396	158	71.48	0.60
d*Go*-g-poly(MMA)	26.54	467	216	68.37	0.53
d*Go*-g-poly(MMA-co-EMA)	51.56	511	428	54.41	0.48
d*Go*-g-poly(MMA-co-EA)	86.32	508	472	51.31	0.43

### Thermal analysis

The TGA of raw, delignified, MMA grafted fibers has been reported earlier (Singha et al., 2014). TGA of raw, delignified, MMA grafted fibers, dGo-g-poly(MMA-co-EMA), and dGo-g-poly(MMA-co-EA) were studied as a function of percentage weight loss vs. temperature and results are shown in Table 3. The initial decomposition temperature (IDT) of the delignified *Grewia optiva* fiber has been found to be 250.13°C, while the final decomposition temperature (FDT) was recorded at 375.39°C. The IDT and FDT for delignified *Grewia optiva* fiber were higher than that of the raw fiber. The grafted sample exhibits two-stage decomposition behavior, IDT and FDT were found to be 261.13 and 432.18°C, respectively, which were much higher than that of the raw and delignified *Grewia optiva* fiber. It may be due to the decomposition of the cellulosic material in the first stage and decomposition of poly MMA in second stage. From the table it is also evident that there has been an increase in the IDT of MMA grafted fiber upon graft copolymerization with different binary monomer mixture. The increase in the IDT of grafted fibers could be attributed to the incorporation of the polymer chains of comonomers on the surface of fiber. Further IDT of fibers with binary mixture of MMA-co-EA was higher than that of other binary monomer mixture which probably may be due to more thermal stability of EA. FDT values for dGo-g-poly(MMA-co-EMA) and dGo-g-poly(MMA-co-EA) were found to be 490.24 and 523.52°C, respectively.

**Table 3:** **Thermogravimetric analysis of grafted and ungrafted samples**.

**Sample name**	**P_g_**	**IDT (°C)**	**FDT (°C)**	**DT (°C) at every 20% weight loss**			**Residual left (%)**
				**20**	**40**	**60**	
Raw fiber	–	241.18	356.38	287.74	326.35	348.77	18.93
Delignified fiber	–	250.13	375.39	302.45	348.31	368.13	18.03
dGo-g-poly(MMA)	26.54	261.13	432.18	294.11	333.62	401.11	13.84
dGo-g-poly(MMA-co-EMA)	51.56	270.23	490.24	304.45	345.65	413.43	7.63
dGo-g-poly(MMA-co-EA)	86.32	273.63	523.52	325.54	357.84	434.84	4.57

### Dye adsorption study

The grafted and ungrafted fiber has been used as adsorbent for removal of methylene blue from water system. At a fixed adsorbent dosage of fibers, the amount of dye adsorbed increased with the concentration of dye. It may be due to the increase in the driving force of the concentration gradient at higher initial dye concentration. It has been revealed that the percentage of adsorption increased initially and then became almost constant. Thus, dye concentration is one of the most important factors controlling the adsorption of dye onto adsorbent. Adsorption is a mass transfer process that can generally be defined as the accumulation of material at the interface between two phases (Table 4). The q_e_ value for grafted fiber was much higher than that of ungrafted fiber.

**Table 4:** **q_*e*_ values for dye adsorption of raw, MMA grafted, MMA-co-EMA, and MMA-co-EA grafted *Grewia optiva* fiber**.

**Dye concentration (mg/L)**	**q_e_ (mg/g)**			
	**Raw fiber**	**dGo-g-poly (MMA)**	**dGo-g-poly (MMA-co-EMA)**	**dGo-g-poly (MMA-co-EA)**
5	2.92	3.07	3.22	3.40
10	3.45	3.78	3.92	4.16
15	4.77	4.82	5.25	5.48
20	7.05	7.35	7.50	7.75
25	7.12	7.46	7.82	7.98
30	7.18	7.64	7.92	8.20

## Conclusion

MWR induced grafting is an effective method for modifying the properties of natural fibers in terms of graft yield, time consumption, and cost effectiveness. The grafting of MMA-co-EMA, MMA-co-EA binary mixtures onto delignified *Grewia optiva* fiber in the presence of ASC/H_2_O_2_ as a redox initiator has been found to have physicochemical, thermal, as well as morphological impact. Although with an increase in grafting, percentage crystallinity and CI decreased, but incorporation of Poly(MMA-co-EMA), and Poly(MMA-co-EA) chains on the backbone polymer resulted in higher acid, base, and thermal resistance properties as compared to those of delignified sample. Moreover, on grafting morphological changes with respect to surface topography have taken place and graft copolymers have been found to exhibit different physical and chemical properties. Therefore, the cellulosic fibers graft copolymerized with vinyl monomers from their binary mixtures have improved properties which can ensure the utilization of these fibers in various industrial applications. Moreover, the removal of methylene dye was found more for grafted samples as compared to that with ungrafted samples.

### Conflict of interest statement

The authors declare that the research was conducted in the absence of any commercial or financial relationships that could be construed as a potential conflict of interest.
